# Butylidenephthalide facilitates contractions via nonspecific binding to receptors in isolated guinea-pig vas deferens

**DOI:** 10.1080/13880209.2019.1619781

**Published:** 2019-06-02

**Authors:** Chung-Hung Shih, Chi-Ming Chen, Wun-Chang Ko

**Affiliations:** a Department of Internal Medicine, Division of Thoracic Medicine, Taipei Medical University Hospital, Taipei, Taiwan;; b School of Respiratory Therapy, College of Medicine, Taipei Medical University, Taipei, Taiwan;; c Department of Medicinal Chemistry, School of Pharmacy, College of Pharmacy, Taipei Medical University, Taipei, Taiwan;; d Department of Pharmacology, School of Medicine, College of Medicine, Taipei Medical University, Taipei, Taiwan

**Keywords:** Ca^2+^ channel blockers, clonidine, noradrenaline, postjunctional K^+^ channel blockade, voltage-dependent Ca^2+^ channels

## Abstract

**Context:** Butylidenephthalide (Bdph) has been reported to inhibit rat uterine contractions, but significantly potentiate the noradrenaline (NA)-induced contractions in guinea-pig vas deferens (GPVDs).

**Objective:** The present study elucidates the binding specificity of Bdph in GPVD to potentiate contractions.

**Materials and methods:** Electrical field stimulation (EFS, supramaximal voltage, 1 ms and 1 Hz) or exogenous NA (50 μM) was applied to the GPVD in Krebs or 1/10 Mg-Tyrode’s solution, respectively. After the clonidine (10 nM)-induced twitch inhibition or the exogenous NA-induced contractions reached a constant, Bdph (50 µM) was added 2 min prior to the subsequent addition of NA (50 µM). Three experiments were performed. In the presence of Bdph (100 μM), the release of NA in the medium and remaining NA content in the tissues were determined after EFS-stimulation.

**Results:** Bdph (100 μM) significantly antagonized the clonidine (10 nM)-induced twitch inhibition from 22.5 ± 2.1 to –11.4 ± 1.6% (*n* = 6) and dibutyryl-cAMP (300 μM) from 25.7 ± 3.2 to 7.9 ± 4.0% (*n* = 8). Bdph (100 μM) significantly increased the electrically stimulated release of NA from 393.0 ± 109.5 to 1000.0 ± 219.1 ng/g (*n* = 6). Bdph (50 μM) potentiated the exogenous NA (50 μM)-induced contractions from 3.0 ± 0.06 to 3.9 ± 0.06 g (*n* = 3), but after washout of Bdph, the response to NA gradually curtailed.

**Discussion and conclusions:** Bdph action may be through the nonspecific binding of the butylidene group to prejunctional α_2_- and postjunctional α_1_-adrenoceptors to reversibly block K^+^ channels, and irreversibly block VDCCs on the smooth muscle cell membrane, respectively.

## Introduction

The rhizome of *Ligusticum chuanxiong* Hort. (Umbelliferae) has been used in China for several thousand years. Its main constituent, butylidenephthalide (Bdph), was proved to be the most active antispasmodic for inhibiting rat uterine contractions induced by prostaglandin F_2α_ (Ko et al. 1977). Bdph has two geometric isomers; *Z*- and *E*-forms. *Z*- and *E*-Bdph were reported to comprise approximately 85% and 15% of Bdph, respectively, in naturally occurring or synthetic products (Ko et al. 1978; Lin et al. 1984). In contrast to *Z*-Bdph, *E*-Bdph competitively and more potently inhibited Ca^2+^-induced contractions in the depolarized (K^+^, 60 mM) guinea pig ileum, suggesting that stereoselectivity occurs in postjunctional voltage-dependent Ca^2+^ channels (VDCCs) (Ko et al. 1997). *Z*-Bdph may inhibit the prejunctional R-type, but not the N-type, VDCCs of cholinergic nerve endings (Chen and Ko 2016). By contrast, the binding of *Z*-Bdph to the nonadrenergic prejunctional VDCCs of the cell membrane was more potent than that of *E*-Bdph in the electrically stimulated prostatic portion of rat vas deferens with stereoselectivity during twitch inhibition (Shih et al. 2016).

In our previous report (Ko 1980), we noted that Bdph (100–250 μM) significantly potentiated the contractions induced by noradrenaline (NA, 1–10 μM) in the guinea-pig vas deferens (GPVDs). Furthermore, Bdph at a higher concentration (500 μM) noncompetitively inhibited NA (10–100 μM)-induced contractions in the tissue. However, it became more sensitive to NA (1–3 μM) compared with the control. Notably, after washout of the tissue exposed to Bdph (500 μM), the potentiation in the contractions induced by NA (1–30 μM) was also observed. The present study elucidates the binding specificity of Bdph in this tissue to potentiate contractions.

## Materials and methods

### Drugs and animals

Bdph (mol. wt., 188.23) was synthesized in accordance with the previously described method (Mowry et al. 1949; Lin et al. 1984) and produced a light-yellow oily substance that is a mixture of 85% trans (*Z*)- and 15% cis (*E*)-isomers. Caffeine sodium benzoate (≥98%), dibutyryl adenosine 3′,5′-cyclic-monophosphate (db-cAMP, ≥97%), imidazole (≥99%) and tetrodotoxin (≥98%) were purchased from Sigma-Aldrich (St. Louis, MO). Other drugs used in this study included aminophylline (Eizai, Tokyo, Japan), clonidine (Boehringer-Ingelheim, Ridgefield, CT) and NA (Breon, NY). The final concentrations of the aforementioned drugs in the medium were expressed in molarity.

Male Hartley guinea-pigs (500–600 g, each) were obtained from the Animal Center of the Ministry of Science and Technology, Taipei, Taiwan. The animals were housed in ordinary cages at 22 ± 1 °C with a humidity of 50–60% under a constant 12 h light/dark cycle and were provided with food and water *ad libitum*. Under a protocol (LAC-74-0032) approved by the Animal Care and Use Committee of Taipei Medical University on 14 October 1985, the GPVD were dissected under anaesthesia (pentobarbital 50 mg/kg, intraperitoneal injection).

### Twitch response test

The vas deferens of guinea-pigs were cut and randomly mounted in 7.5 mL of Krebs solution, oxygenated with 95% O_2_–5% CO_2_ and maintained at 37 °C. Along the tissues with an initial tension of 0.5 g, parallel platinum wires were set. Electrical field stimulation (EFS, supramaximal voltage, 1 ms and 1 Hz) was derived from a Grass S-88 stimulator and applied to the tissues for 6 s as a train each time. The train rate was one per minute. The twitch responses were isometrically recorded using a polygraph (Gould RS3200, Valley View, OH) and proved to be blocked by tetrodotoxin (1 μM), suggesting that the twitch responses were neurogenic. The Krebs solution consisted of the following composition (mM): NaCl 119, KCl 4.7, CaCl_2_ 2.5, NaH_2_PO_4_ 1.2, NaHCO_3_ 25 and dextrose 11. After the twitch response reached a constant, clonidine (0–300 nM), an α_2_-adrenoceptor agonist, or NA (0–10 μM) was cumulatively added when the effect of the agonist at each concentration reached a constant. The log concentration–response curves of clonidine and NA were constructed.

### Antagonism against clonidine-induced twitch inhibition

After the twitch inhibition using clonidine (10 nM) reached a constant, each test drug, namely Bdph, aminophylline, db-cAMP, caffeine and imidazole, was cumulatively added.

### Contractile response by an exogenous agonist

The vas deferens of the guinea-pigs were cut and mounted in 7.5 mL of 1/10 Mg-Tyrode’s solution with an initial tension of 0.5 g, oxygenated with 95% O_2_–5% CO_2_ and maintained at 37 °C, in accordance with a previously described method (Ko 1980). The contractile responses were isometrically recorded using a polygraph (Gould RS3200, Valley View, OH). After NA (50 μM)-induced contractions reached a constant, Bdph was added 2 min prior to the subsequent addition of NA. When the contraction reached the maximum and subsequently stopped the recording, the tissue was washed using 1/10 Mg-Tyrode’s solution three times. The addition of NA and washout was repeated until the contractile response disappeared. The 1/10 Mg-Tyrode’s solution consisted of the following composition (mM): NaCl 137, KCl 2.7, MgCl_2_ 0.1, NaH_2_PO_4_ 0.42, CaCl_2_ 1.8, NaHCO_3_ 11.9 and dextrose 5.55. The 1/10 Mg-Tyrode’s solution was the same as normal Tyrode’s solution except that 0.1 mM MgCl_2_ instead of 1.0 mM MgCl_2_ was included to increase the sensitivity to NA (Ko 1980).

### Determination of NA release

Similar to the experiment of twitch response test, the vas deferens of the guinea-pigs were mounted in 7.5 mL of Krebs solution containing bovine serum albumin (2.5%), phenoxybenzamine (50 μM) and sodium ascorbate (0.4 mg/mL), oxygenated using 95% O_2_–5% CO_2_ and maintained at 37 °C. After equilibrium, the tissues were incubated using Bdph (100 μM) as a test or its vehicle (0.4% ethyl alcohol) as a control. After 30 min-incubation, the spontaneous release of NA in the medium was collected and determined. Subsequently, the EFS (supramaximal voltage, 1 ms and 1 Hz) derived from a Grass S-88 stimulator was applied to the tissues for 1 h. After stimulation, the release of NA in the medium and remaining NA content in the tissues was determined. The determination was performed in accordance with a previously described method (Anton and Sayre 1962).

### Statistical analysis

All results are presented as the mean ± standard error of the mean (SEM) (*n*), where *n* is the number of experiments. Difference between two values was determined using Student’s paired or unpaired *t*-test. Differences of *p* < 0.05 were considered statistically significant.

## Results

### Clonidine or NA concentration dependently induced twitch inhibitions

**Figure 1.**
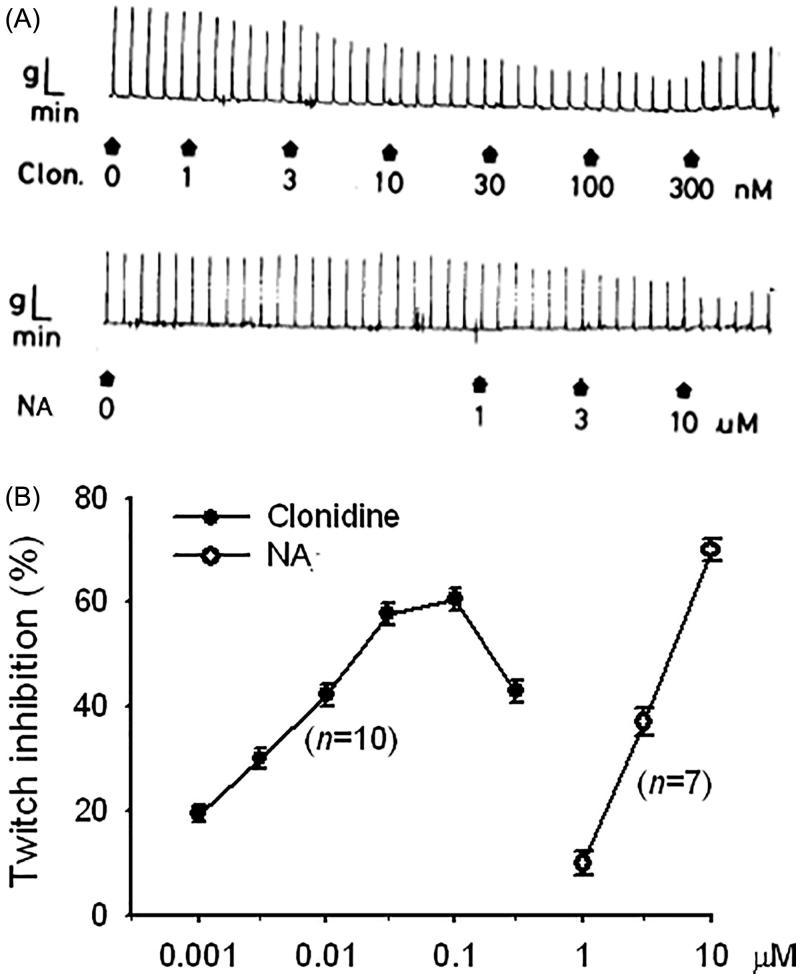
Tracing graphs (A) of clonidine (Clon., upper panel)- and noradrenaline (NA, lower panel)-induced twitch inhibition, and their log concentration-inhibition relationships (B) in the electrically stimulated guinea-pig vas deferens. Each point represents the mean ± SEM, and *n* is the number of experiments. Drugs were added at the arrow shown in the tracing graphs.

The twitch responses were inhibited by clonidine or NA in a concentration-dependent manner as shown in tracing graphs (Figure 1(A)) and a statistical graph (Figure 1(B)).

### Antagonism against clonidine-induced twitch inhibition

**Figure 2.**
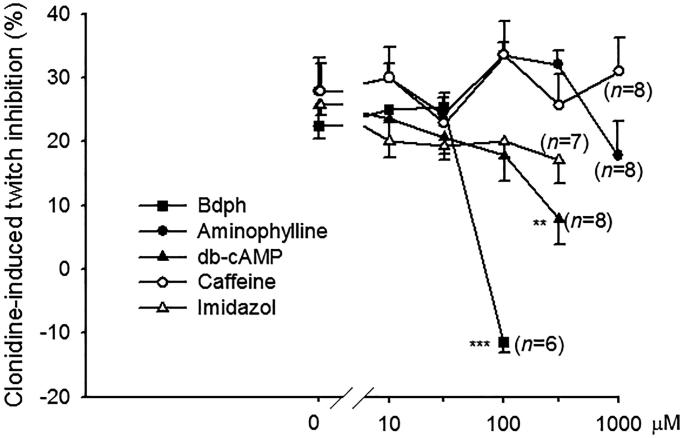
Antagonistic effect of butylidenephthalide (Bdph) or dibutyryl cAMP (db-cAMP) on clonidine (10 nM)-induced twitch inhibition in the electrically stimulated guinea-pig vas deferens. Each point represents the mean ± SEM, and *n* is the number of experiments. **p < 0.01, ***p < 0.001, compared with their controls (0 μM, vehicle) by Student’s paired *t*-test.

The clonidine (10 nM)-induced inhibition was significantly antagonized by Bdph (100 μM) from 22.5 ± 2.1% to –11.4 ± 1.6% (*n* = 6) and db-cAMP (300 μM) from 25.7 ± 3.2% to 7.9 ± 4.0% (*n* = 8). However, the clonidine (10 nM)-induced inhibition was not affected by phosphodiesterase (PDE) inhibitors, such as caffeine and aminophylline, even at a concentration of 1 mM, or by the PDE activator, imidazole even at a concentration of 300 μM (Figure 2).

### Bdph potentiated exogenous NA-induced contractions and faded after washout

**Figure 3.**
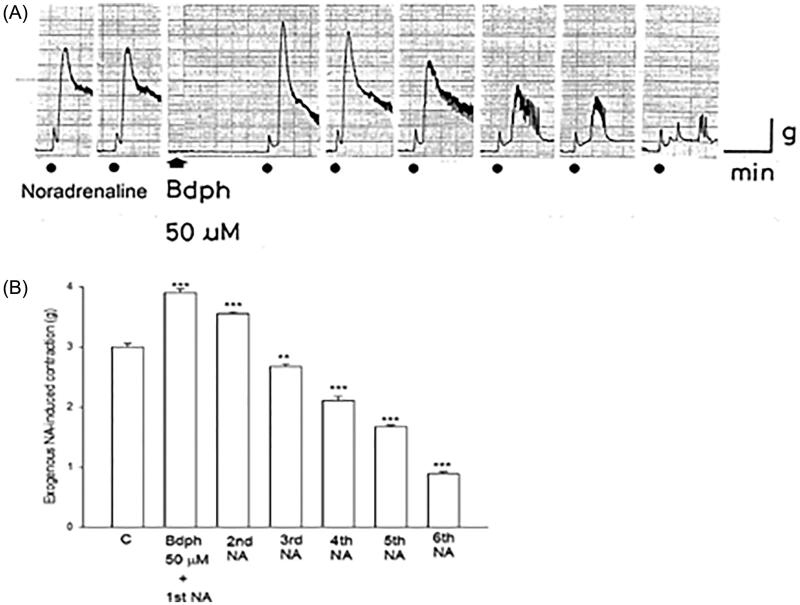
(A) Tracing graph of Butylidenephthalide (Bdph) potentiated the exogenous noradrenaline (NA, 50 μM)-induced contraction of the guinea-pig vas deferens. Washout of Bdph was performed after each contraction and subsequently added NA (50 μM) from 2nd to 6th. The dots mark where NA was added, and the arrow marks where Bdph was added. (B) Each column represents the mean ± SEM, and the number of experiments was 3. **p < 0.01, ***p < 0.001, compared with its control (C, 0.2% ethyl alcohol) by Student’s unpaired *t*-test.

Bdph (50 μM) potentiated the exogenous NA (50 μM)-induced contractions from 3.0 ± 0.06 to 3.9 ± 0.06 g (*n* = 3), and subsequently gradually curtailed the contractions after each washout until no response to NA was observed (Figure 3). Three similar results were observed in this study.

### Bdph facilitated the electrically stimulated release of NA

**Figure 4.**
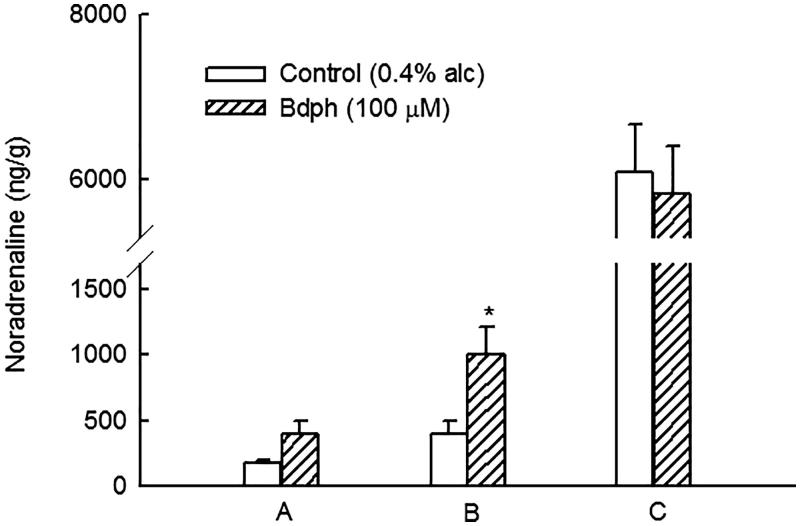
Butylidenephthalide (Bdph) augmented the electrically stimulated release of NA in the guinea-pig vas deferens. (A) Spontaneous release of NA in the medium; (B) electrically stimulated release of NA in the medium; (C) NA content in the vas deferens after stimulation. Each column represents the mean ± SEM, and the number of experiments was 6. *p < 0.05 compared with its control (0.4% ethyl alcohol) by Student’s unpaired *t*-test.

Bdph (100 μM) significantly increased the electrically stimulated release from 393.0 ± 109.5 to 1000.0 ± 219.1 ng/g (*n* = 6), but not the spontaneous release, of NA from the tissue into the medium (Figure 4). The remaining NA content in the tissue was uninfluenced by Bdph compared with the control (Figure 4).

## Discussion

The EFS of sympathetic nerves in GPVD results in a Ca^2+^ influx through VDCCs on the prejunctional membrane and in a contraction with two distinct components. The twitch or phasic component is transient and insensitive to nifedipine, an l-type Ca^2+^ channel blocker, whereas the secondary tonic component is sustained for the duration of stimulation and sensitive to nifedipine (Swedin 1971; Kaplita and Triggle 1983). The neurogenic transmitters are NA and adenosine triphosphate (ATP), a cotransmitter of NA that was confirmed to be purine (Westfall et al. 1978). The released NA acts on the postjunctional α_1_-adrenoceptor to induce a nifedipine-insensitive twitch contraction (Minneman et al. 1988), and ATP acts on the ligand-gated P2X_1_-receptors to evoke a contraction (Liang et al. 2000; Mulryan et al. 2000), which was demonstrated to be blocked by nifedipine (Cleary et al. 2003). However, some released NA transmitters are uptaken across the prejunctional membrane into the vesicles, and some act on the prejunctional α_2_-adrenoceptor to inhibit NA release for feedback regulation. In the present studies, NA or clonidine, an α_2_-adrenoceptor agonist (Maze et al. 1988), acted on prejunctional α_2_-adrenoceptors and inhibited NA release and thus caused concentration-dependent twitch inhibition (Figure 1). The inhibition of adenylyl cyclase activity was the earliest effect observed in α_2_-receptor activation; however, in some systems, the enzyme is actually stimulated by α_2_-receptors either by protein G_i_βγ subunits or by the weak direct stimulation of protein G_s_ (Westfall and Westfall 2011). Thus, the clonidine-induced twitch inhibition in the isolated GPVD might be through adenylyl cyclase stimulation, which subsequently increases the cAMP content, by which cAMP-dependent protein kinase is activated. Thus, it results in increased calcium extrusion from the intracellular space and uptake to the endoplasmic reticulum and finally reduces intracellular calcium ([Ca^2+^]*_i_*) concentration. The activation of prejunctional α_2_-adrenoceptors can inhibit VDCCs through protein G_o_ mediation and reduce [Ca^2+^]*_i_* (Westfall and Westfall 2011). Ultimately, it causes the twitch inhibition through either method. By contrast, the inhibition of prejunctional α_2_-adrenoceptors increases [Ca^2+^]*_i_* and causes twitch facilitation. However, the clonidine-induced twitch inhibition was affected neither by PDE inhibitors, such as caffeine and aminophylline, nor by the PDE activator imidazole (Roussinov et al. 1976). The influence of the PDE activator and inhibitors on the twitch inhibition was insufficient for measurement. Another possibility may be that the concentration that was used was too low because caffeine at 3 and 10 mM was reported to increase the amplitude of the first few excitatory junction potentials (EJPs) of smooth muscle cells in the GPVD in each train and reduce the extent of facilitation and the amplitude of fully facilitated EJPs in the electrically stimulated hypogastric nerve (Ziogas et al. 1995). By contrast, the clonidine-induced twitch inhibition was significantly antagonized and non-competitively by Bdph or db-cAMP at a high concentration of 100 or 300 μM, respectively. Notably, they are with a butylidene group or two butyryl groups, respectively. We reported that even dibutyl phthalate inhibited the prostaglandin F_2α_-induced contraction in the rat uterus as a nonspecific antispasmodic, although phthalic acid had no effects on the contraction (Ko et al. 1977). The butyl, butylidene or butyryl group enhances the lipophilic property of these compounds to penetrate the membranes of smooth muscle cells. In human embryonic kidney cells, 1-dodecanol and farnesol were reported to exhibit a significantly lower blocking affinity for N-type Ca^2+^ channels, and dodecylamine was reported to block those with the highest affinity, suggesting that the functional group is a critical determinant (Beedle and Zamponi 2000). Thus, the antagonism of Bdph or db-cAMP against clonidine-induced twitch inhibition may be through the nonspecific inhibition of the prejunctional α_2_-adrenoceptor membrane to reduce the binding of clonidine to these receptors. This new finding is so different from that of our previous report (Shih et al. 2018). Prejunctional α_2_-adrenoceptors also activate protein G-gated K^+^ channels, resulting in membrane hyperpolarization (Westfall and Westfall 2011). Thus, clonidine-induced twitch inhibition in the isolated GPVD may be through the opening of K^+^ channels and membrane hyperpolarization. Furthermore, Bdph, similar to 4-aminopyridine (Hsu et al. 2014), a voltage-gated K^+^ channel blocker (Choquet and Korn 1992) blocked voltage-gated K^+^ channels to induce membrane depolarization and facilitation. In the present study, Bdph (100 μM) significantly increased the electrically stimulated release of NA from the tissue into the medium. This effect may be because of the blockade of voltage-gated K^+^ channels. Bdph was reported to antagonize cromakalim-induced guinea-pig tracheal relaxation (Hsu et al. 2014), in which cromakalim was an ATP-dependent K^+^ channel opener for smooth muscle cells (Escande et al. 1988).

In the present studies, facilitating responses to exogenous NA were determined before and after the first washout of Bdph, suggesting that the nonspecific binding of Bdph (50 µM) on the postjunctional α_1_-adrenoceptor causes the effector cell membrane to become more excitable (Figure 3). The increased excitability seems to be related to the K^+^ channel blocking effects of Bdph. Recently, we reported that Bdph similarly to 4-aminopyridine, a blocker of the Kv 1 family of K^+^ channels, enhanced the baseline tension of the guinea-pig trachea (Hsu et al. 2014). The binding of Bdph to the K^+^ channels of the smooth muscle cell membrane seems partially reversible, but the binding of Bdph to VDCCs seems irreversible. Thus, the facilitating responses of Bdph to exogenous NA were observed before and after the first washout of Bdph. However, the inhibiting effects of Bdph to NA gradually faded, ultimately, no response to NA was observed, suggesting that its binding to VDCCs is irreversible (Figure 3).

## Conclusions

Bdph (100 µM) antagonized clonidine-induced twitch inhibition and facilitated NA release from vesicles in the electrically stimulated GPVD. This effect may be because of the nonspecific binding of the butylidene group to the prejunctional α_2_-adrenoceptors, which blocked voltage-gated K^+^ channels. Bdph (50 µM) potentiated exogenous NA-induced contraction of the tissue may be because of this nonspecific binding on the postjunctional α_1_-adrenoceptors, which blocks K^+^ channels on the smooth muscle cell membrane. The nonspecific binding of Bdph to the postjunctional VDCCs of α_1_-adrenoceptors seems to be irreversible, but that to the K^+^ channels seems to be partially reversible.
